# Integrative Computational Approach Revealed Crucial Genes Associated With Different Stages of Diabetic Retinopathy

**DOI:** 10.3389/fgene.2020.576442

**Published:** 2020-11-12

**Authors:** Nidhi Kumari, Aditi Karmakar, Saikat Chakrabarti, Senthil Kumar Ganesan

**Affiliations:** ^1^Department of Structural Biology & Bioinformatics, CSIR-Indian Institute of Chemical Biology, Kolkata, India; ^2^CSIR-IICB Translational Research Unit of Excellence (TRUE), Kolkata, India; ^3^Academy of Scientific and Innovative Research (AcSIR), Ghaziabad, India

**Keywords:** diabetic retinopathy, integrative approach, candidate genes, hub genes, early genes, biomarker

## Abstract

The increased incidence of diabetic retinopathy (DR) and the legacy effect associated with it has raised a great concern toward the need to find early diagnostic and treatment strategies. Identifying alterations in genes and microRNAs (miRNAs) is one of the most critical steps toward understanding the mechanisms by which a disease progresses, and this can be further used in finding potential diagnostic and prognostic biomarkers and treatment methods. We selected different datasets to identify altered genes and miRNAs. The integrative analysis was employed to find potential candidate genes (differentially expressed and aberrantly methylated genes that are also the target of altered miRNAs) and early genes (genes showing altered expression and methylation pattern during early stage of DR) for DR. We constructed a protein-protein interaction (PPI) network to find hub genes (potential candidate genes showing a greater number of interactions) and modules. Gene ontologies and pathways associated with the identified genes were analyzed to determine their role in DR progression. A total of 271 upregulated-hypomethylated genes, 84 downregulated-hypermethylated genes, 11 upregulated miRNA, and 30 downregulated miRNA specific to DR were identified. 40 potential candidate genes and 9 early genes were also identified. PPI network analysis revealed 7 hub genes (number of interactions >5) and 1 module (score = 5.67). Gene ontology and pathway analysis predicted enrichment of genes in oxidoreductase activity, binding to extracellular matrix, immune responses, leukocyte migration, cell adhesion, PI3K-Akt signaling pathway, ECM receptor interaction, etc., and thus their association with DR pathogenesis. In conclusion, we identified 7 hub genes and 9 early genes that could act as a potential prognostic, diagnostic, or therapeutic target for DR, and a few early genes could also play a role in metabolic memory phenomena.

## Introduction

Diabetic retinopathy (DR), one of the major microvascular complications of diabetes, is affecting approximately 34.6% of diabetic individuals and has become the greatest threat to vision (Yau et al., 2012). It starts with few microaneurysms and dot hemorrhages during its initial stage, i.e., Non-Proliferative Diabetic Retinopathy (NPDR), and progresses to the sight-threatening stage called Proliferative Diabetic Retinopathy (PDR). Various structural abnormalities like thickening of basement membrane, pericyte loss, breakdown of blood-retinal barrier, etc., are also associated with DR. Studies have shown that neurodegeneration of ganglion cells is the most initial event of DR pathogenesis, which starts even before the formation of microaneurysm and dot hemorrhages (Barber and Baccouche, 2017). DR remains asymptomatic in its initial stages; however, symptoms like dark string floating in the visual field, blurred vision, etc., start appearing as the disease progresses and, if not treated, may end with loss of vision. DR is associated with alteration in various metabolic pathways like polyol pathway, hexosamine pathway, protein kinase C (PKC) pathway, accumulation of advanced glycosylation end products (AGEs), etc., which aggravates oxidative stress and inflammatory responses and thus the disease condition. Organelles like mitochondria and endoplasmic reticulum are highly affected in DR conditions. The alterations in the expression level, methylation pattern, and several other genetic and epigenetic modifications of various genes, especially those related to oxidative stress, inflammation, and angiogenesis, drive the pathogenesis and progression of DR by affecting multiple molecular pathways and functions (Wong et al., 2016).

Various epigenetic modifications such as DNA methylation, histone modifications, microRNA (miRNA), etc., occurring during the early stage of diabetes do not only regulate the expression of various genes but are also responsible for the metabolic memory phenomena (deleterious effect induced by prior glycemic exposure regardless of later glycemic control) associated with diabetes (Mishra and Kowluru, 2016; Kumari et al., 2020). This triggers the need for developing early diagnosis and treatment methods. Further, limitations of available treatments like its cost-effectiveness, variation in responses from patients to patients, unavailability in remote areas, etc., have raised the concern for better diagnosis and treatment strategies.

Almost all the complications are the result of and also lead to significant alterations in the expression pattern of various genes. There are various epigenetic, genetic, as well as other modifications responsible for such alterations. Identifying aberrantly expressed genes, miRNAs, altered methylation, and acetylation patterns are the first and the most critical step toward understanding the mechanisms by which the disease progresses, and this can be further used in finding the potential prognostic and treatment methods and also in identifying various biomarkers. Microarray profiling of genes is an emerging tool to screen significantly altered genes or miRNAs present in a specific disease condition. This tool can be exploited to identify candidate genes and diagnostic and prognostic biomarkers for a particular disease (Tarca et al., 2006; Moradifard et al., 2018).

The individual analysis of the array data is not very reliable and precise. This limitation can be overcome to some extent by overlapping usage of various relevant datasets (Curran and Hussong, 2009; S. Kim and Park, 2016). In the present study, integrative analysis of gene expression profiling microarray data, gene methylation profiling microarray data, and miRNA expression profiling microarray data were performed and various bioinformatics tools were utilized to find potential candidate genes and genes altered during early stage of DR, which may be used as a diagnostic or prognostic biomarkers specific for DR. Protein-protein interaction network construction, pathways, and functional analysis of identified genes were performed to investigate the molecular mechanisms associated with DR.

## Materials and Methods

### Microarray Data and Processing

The data of gene expression profiling, gene methylation profiling, and miRNA expression profiling were obtained from Gene Expression Omnibus (GEO) datasets available at National Center for Biotechnology Information (NCBI)^1^. The first preference was given to datasets containing human samples for the specific disease followed by datasets containing greater number of samples and then the datasets from recent studies. The statistical significance, normalization, and quality of data present in datasets were ensured from the literature containing respective studies. We employed GEO2R tool^1^ to download all the raw data (*p*-value adjusted to false discovery rate [FDR]) of a particular group of samples present in the selected dataset and identified the differentially expressed genes (DEGs), differentially methylated genes (DMGs), or differentially expressed miRNAs. In order to identify genes altered during early stage of DR, separate comparisons for PDR and NPDR were made from DR datasets containing PDR and NPDR samples.

The diabetic retinopathy (DR) gene expression profiling dataset GSE60436 (platform: GPL6884 Illumina HumanWG-6 v3.0 expression BeadChip) consisted of total 9 human samples (Japanese population) out of which 3 were taken from the normal retina and 6 from the fibrovascular membrane (FVM) of proliferative diabetic retinopathy (PDR) patients. The samples from PDR patients were grouped into active FVM (3 samples) and inactive FVM (3 samples) on the basis of presence or absence of neovascularization (NV) in the FVM, respectively (Ishikawa et al., 2015). To identify DEGs, we performed two sets of comparison: first, normal retina vs. inactive FVM (A), and second, normal retina vs. active FVM (B), with cut-off of *p*-value < 0.05 and absolute log fold change value (|log FC|) ≥ 1.5. However, we merged the data of both sets (A + B) as both contained the samples from PDR patients.

The diabetic retinopathy (DR) gene methylation profiling dataset GSE57362 (platform: GPL13534 Illumina Human Methylation450 BeadChip [HumanMethylation450_15017482]) consisted of total 265 human samples (Spanish population) out of which 8 were from normal neuroretina, 8 were from neuroretina of non-proliferative diabetic retinopathy (NPDR) patients, 9 were from FVM of DR patients, and the rest were from patients suffering from other ocular diseases (Berdasco et al., 2017). Here also two sets of comparison were performed: first, normal neuroretina vs. neuroretina of NPDR with |log FC| > = 0.2 (G), and second, normal neuroretina vs. FVM of PDR with |log FC| > = 0.5 (H), to identify DMGs with *p*-value < 0.05. Here, we have set less threshold for |log FC| with the assumption that fold change depends on many factors like type of study performed, stage of disease at which sample was collected, methods used to perform the study, etc. Hence we assumed that the fold change in methylation profiling study might be far lower than that in the expression profiling study (Maag et al., 2017; Raman et al., 2018; Abdulrahim et al., 2019; He et al., 2019; Yang et al., 2019).

The diabetic retinopathy (DR) miRNA expression profiling dataset GSE140959 (platform: GPL16384 [miRNA-3] Affymetrix Multispecies miRNA-3 Array) consisted of total 73 human samples (from United States) of macular hole (MH), PDR, and NPDR patients from aqueous humor (10 MH, 4 NPDR, 10 PDR), vitreous humor (10 MH, 4 NPDR, 10 PDR), and plasma (10 MH, 4 NPDR, 11 PDR) (Smit-McBride et al., 2020). For this dataset a total of 4 comparisons were made with *p*-value < 0.05 and |log FC| > = 1.5: first, aqueous and vitreous humor of normal vs. NPDR (C’); second, aqueous and vitreous humor of normal vs. PDR (D’); third, plasma of normal vs. NPDR (E’); and fourth, plasma of normal vs. PDR (F’). Plasma samples were compared separately with the thought of identifying any circulatory biomarker.

The diabetic nephropathy (DN) gene expression profiling dataset GSE1009 (platform: GPL8300 [HG_U95Av2] Affymetrix Human Genome U95 Version 2 Array) consisted of total 6 human samples (from Netherlands) out of which 3 were from the glomeruli of normal kidney and 3 from the glomeruli obtained from the diabetic nephropathy kidney (Baelde et al., 2004), and the DEGs were identified by performing comparison between glomeruli of normal kidney vs. glomeruli of DN kidney (1) with cut-off of *p*-value < 0.05 and |log FC| ≥ 1.5.

The diabetic nephropathy (DN) miRNA expression profiling dataset GSE51674 (platform: GPL10656 Agilent-029297 Human miRNA Microarray v14 Rev.2 [miRNA ID version]) consisted of total 16 human samples (from Italy) out of which 4 were from kidney of healthy control, 6 were from kidney of DN patients, and 6 from kidney of diabetic patients with membranous nephropathy (Conserva et al., 2019), and comparison was made between kidney tissue sample of normal vs. DN individuals (3) with *p*-value < 0.05 and |log FC| ≥ 1.5.

The diabetic foot ulcer (DFU) gene expression profiling dataset GSE80178 (platform: GPL16686 [HuGene-2_0-st] Affymetrix Human Gene 2.0 ST Array [transcript (gene) version]) consisted of total 12 human samples (from United States) out of which 6 were of diabetic foot ulcer, 3 of diabetic foot skin, and 3 of non-diabetic foot skin (Ramirez et al., 2018), and DEGs were identified from comparison between non-diabetic foot skin vs. diabetic foot ulcer (2) with cut-off value of *p*-value < 0.05 and |log FC| ≥ 1.5.

The diabetic foot ulcer (DFU) miRNA expression profiling dataset GSE84971 (platform: GPL17537 nCounter Human miRNA Expression Assay, V2) consisted of total 6 human foot fibroblast samples (from United States) out of which 3 were from diabetic foot ulcer and 3 from non-diabetic foot (Liang et al., 2016), and the comparison between foot fibroblast samples of non-diabetic foot vs. diabetic foot ulcer (4) was made with *p*-value < 0.05 and |log FC| ≥ 1.5.

**FIGURE 1:**
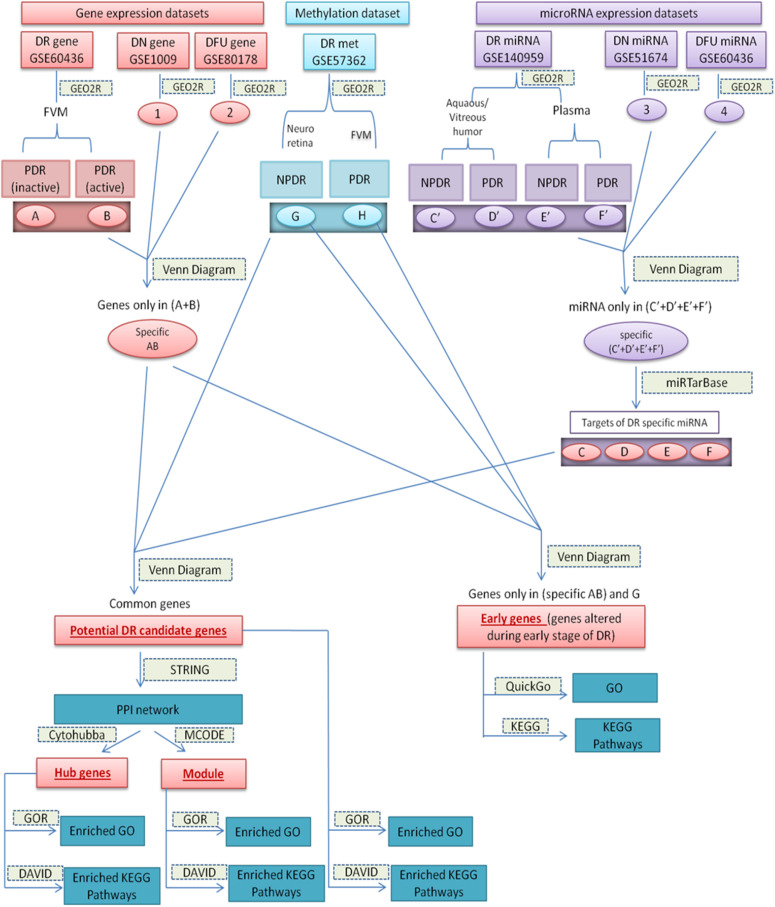
Diagrammatic representation of overall work-flow. **(A)**: (DEGs in inactive FVM of PDR); **(B)**: (DEGs in active FVM of PDR); **(G)**: (DMGs in neuroretina of NPDR); **(H)**: (DMGs in FVM of PDR); **(1)**: (DEGs in glomeruli of DN kidney); **(2)**: (DEGs in DFU); **(3)**: (differentially expressed miRNAs in kidney tissue of DN); **(4)**: (differentially expressed miRNAs in foot fibroblast sample of DFU); **(C’)**: (differentially expressed miRNAs in Aqueous and vitreous humor of NPDR); **(D’)**: (differentially expressed miRNAs in Aqueous and vitreous humor of PDR); **(E’)**: (differentially expressed miRNAs in plasma of NPDR); **(F’)**: (differentially expressed miRNAs in plasma of PDR); **(Specific AB)**: (DEGs specific for DR); **(Specific C’–F’)**: (differentially expressed miRNAs specific for DR); **(C–F)**: (targets of DR specific differentially expressed miRNA).

Datasets and the sets of comparison are summarized in Table 1, and the overall work-flow of the study is summarized in Figure 1.

**TABLE 1:** Datasets and the set of comparisons.

Dataset	Comparisons	No. of sample control/diseased	Platform	References
GSE60436 (DR_mRNA)	Normal retina vs. inactive FVM of PDR (A) Normal retina vs. active FVM of PDR (B)	3/3 3/3	GPL6884	Ishikawa et al., 2015
GSE57362 (DR_methylation)	Normal neuroretina vs. neuroretina of NPDR (G) Normal neuroretina vs. FVM of PDR (H)	8/8 8/9	GPL13534	Berdasco et al., 2017
GSE140959 (DR_miRNA)	Aqueous and vitreous humor of normal vs. NPDR (C’) Aqueous and vitreous humor of normal vs. PDR (D’) Plasma of normal vs. NPDR (E’) Plasma of normal vs. PDR (F’)	10/4 and 10/4 10/10 and 10/10 10/4 10/11	GPL16384	Smit-McBride et al., 2020
GSE1009 (DN_mRNA)	Glomeruli of normal kidney vs. DN kidney (1)	3/3	GSL8300	Baelde et al., 2004
GSE51674 (DN_miRNA)	Kidney tissue sample of normal vs. DN (3)	4/6	GPL10656	Conserva et al., 2019
GSE80178 (DFU_mRNA)	Non-diabetic foot skin vs. DFU (2)	3/6	GPL16686	Ramirez et al., 2018
GSE84971 (DFU_miRNA)	Foot fibroblast sample of non-diabetic foot vs. DFU (4)	3/3	GPL17537	Liang et al., 2016

### Determination of Specific and Overlapping Genes and miRNAs

We got two sets of data from each comparison [upregulated (log FC ≥ 1.5) and downregulated (log FC ≤ −1.5) from expression data and hypermethylated (log FC ≥ 0.2 for NPDR and log FC ≥ 0.5 for PDR) and hypomethylated (log FC ≤ −0.2 for NPDR and log FC ≤ −0.5 for PDR) from methylation data]. The specific and overlapping genes or miRNAs were determined using online software Draw Venn Diagram^1^.

#### DR Specific Genes and miRNAs

We performed stepwise comparisons. Initially, to find DR specific aberrantly expressed genes (specific AB) and DR specific aberrantly expressed miRNAs [specific (C’+D’+E’+F’)], we compared differentially expressed genes or miRNAs data of DR with that of DN and DFU (Figure 1) and excluded all those genes and miRNAs that were not exclusively present in DR from further analysis.

#### Potential DR Candidate Genes

We assumed potential DR candidate genes as the genes that showed altered expression as well as methylation pattern and were also the target of altered miRNAs. To identify potential candidate genes for DR, we compared DR specific differentially expressed genes (specific AB), differentially methylated genes (G+H), and targets of the DR specific altered miRNA (C+D+E+F) (Figure 1). The genes that were common among all the three groups were considered as potential candidate genes for DR. To find the targets of altered miRNA, we used miRTarBase^2^ and chose the targets on the basis of strong experimental evidence such as Reporter assay, Western blot, and qPCR.

#### Genes Involved in Early Stage of DR

The criteria for choosing early genes, i.e., the genes involved in an early stage of DR, was to find genes that show differential expression and aberrant methylation pattern in the early stage (NPDR) of DR. So, we compared DR specific differentially expressed genes (specific AB), differentially methylated genes in NPDR (G), and differentially methylated genes in PDR (H). Genes that were present in (specific AB) and (G) were considered as the genes altered during early stage of the disease and can be targeted for early diagnosis and treatment (Figure 1).

### PPI Network Construction, Hub Gene, and Module Identification

We considered hub genes as those potential candidate genes of DR that possess a large number of interactions. Search Tool for the Retrieval of Interacting Genes (STRING) database is one of the most familiar tools to determine the known and predicted interactions among a set of proteins. STRING version 11.0^3^ was used to construct the interaction network between potential candidate genes with sources of interactions including experiments, databases, text mining, co-occurrence, co-expression, and protein homology. A high confidence cut off ≥ 0.7 of minimum interaction score was used to extract the interactions. With the help of Molecular Complex Detection (MCODE) (Bader and Hogue, 2003) and CytoHubba (Chin et al., 2014) applications of Cytoscape (Shannon et al., 2003) we determined highly interconnected clusters or module and hub genes, respectively, present in our PPI networks (Figure 1).

### Gene Ontology and Pathway Analysis

Though gene ontology (GO) provides various biological processes, molecular functions, and sub-cellular localizations of genes, it doesn’t contain any information about the pathways that are associated with the genes. Various subsets of GO and pathways are interdependent and interconnected with each other, so to understand the mechanisms by which a gene works, it is necessary to determine various gene ontologies along with their associated pathways. In this study, gene ontology and pathway analysis were performed for potential DR candidate genes, genes altered during the early stage of DR, i.e., NPDR stage, hub genes, and genes present in the interconnected module. Gene Ontology Resource (GOR)^3^ and Database for Annotation Visualization and Integrated Discovery (DAVID)^4^ are among the most well-known tools to perform gene enrichment analysis. The enrichment analysis of Gene Ontology (GO) (GO biological process complete and GO molecular function complete) was performed using GOR database while that of KEGG pathways was performed using DAVID database with cutoff value of FDR *p* < 0.05. Further, to go into the details of each individual genes present in the hub genes and early genes of DR, we determined the GO and KEGG pathways for each of those genes separately using QuickGo^5^ and KEGG^6^ databases (Figure 1).

## Results

### Identification of Altered Genes and miRNAs in DR

The GEO2R analysis of different gene expression profiling datasets identified total 743 upregulated and 971 downregulated genes in DR, 855 upregulated and 408 downregulated genes in DN, 353 upregulated and 864 downregulated genes in DFU, respectively. In the case of gene methylation profiling of DR dataset total 81 hypermethylated genes in NPDR, 83 hypomethylated genes in NPDR, 584 hypermethylated genes in PDR, and 3699 hypomethylated genes in PDR were identified. The miRNA expression profiling of different datasets identified total 11 upregulated and 30 downregulated miRNA in DR, 126 upregulated and 35 downregulated miRNA in DN, 27 upregulated and 1 downregulated miRNA in DFU, respectively (Supplementary Table 1).

### Identification of Potential DR Candidate Genes

#### DR Specific Genes and miRNAs

**FIGURE 2:**
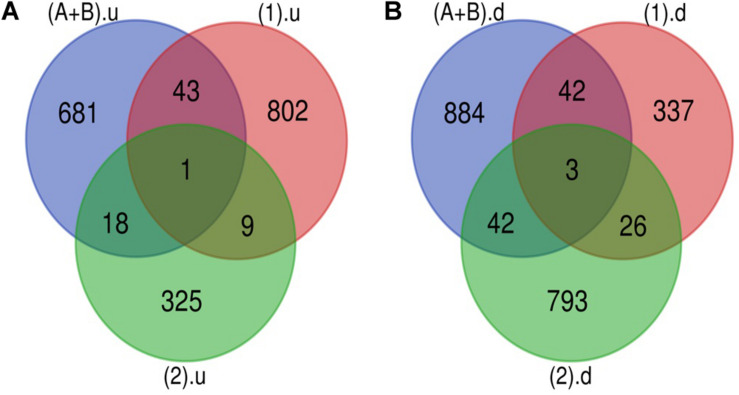
Genes specific for DR. **(A)** Up-regulated genes: total 743 (681 + 18 + 1 + 43) up-regulated genes are present in DR out of which 681 are uniquely present in DR cases. **(B)** Down-regulated genes: total 971 (884 + 42 + 3 + 42) down-regulated genes are present in DR out of which 884 are uniquely present in DR cases (A+B).u: (up-regulated genes in inactive and active FVM of DR); (1).u: (up-regulated genes in DN); (2).u: (up-regulated genes in DFU); (A+B).d: (down-regulated genes in inactive and active FVM of DR); (1).d: (down-regulated genes in DN); (2).d: (down-regulated genes in DFU).

**FIGURE 3:**
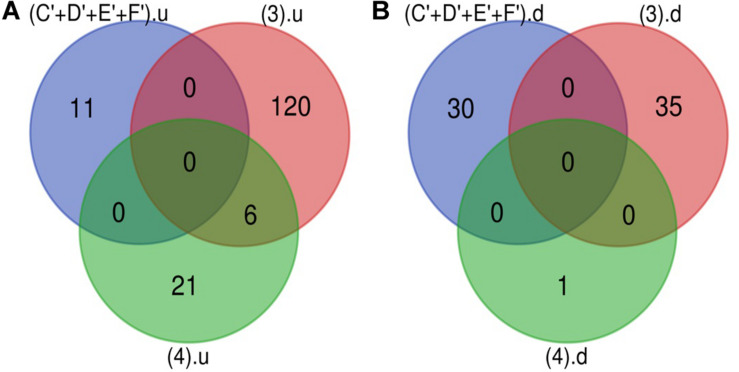
microRNA specific for DR. **(A)** Upregulated miRNA: 11 up-regulated miRNAs are uniquely present in DR cases. **(B)** Downregulated miRNA: 30 down-regulated miRNAs are uniquely present in DR cases (C’+D’+E’+F’).u: (up-regulated miRNA in DR); (3).u: (up-regulated miRNA in DN); (4).u: (up-regulated miRNA in DFU); (C’+D’+E’+F’).d: (down-regulated miRNA in DR); (3).d: (down-regulated miRNA in DN); (4).d: (down-regulated miRNA in DFU).

**FIGURE 4:**
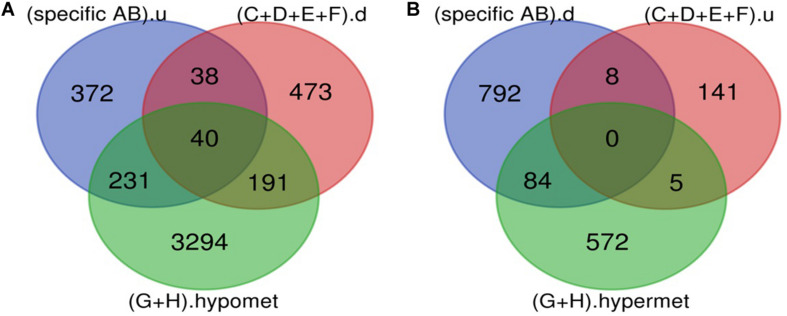
Potential DR candidate genes. **(A)** Upregulated genes_Down-regulated miRNA targets_Hypomethylated genes: 40 up-regulated and hypomethylated genes were also the target of DR specific down-regulated miRNA. **(B)** Down-regulated genes_Up regulated miRNA targets_Hypermethylated genes: 0 down-regulated and hypermethylated were the target of DR specific up-regulated miRNA (specific AB).u: (up-regulated genes specific for DR); (C+D+E+F).d: (targets of DR specific down-regulated miRNA); (G+H).hypomet: (hypomethylated genes in NPDR and PDR); (specific AB).d: (down-regulated genes specific for DR); (C+D+E+F).u: (targets of DR specific up-regulated miRNA); (G+H).hypermet: (hypermethylated genes in NPDR and PDR).

The Venn diagram revealed various specific and overlapping genes. Comparing gene and miRNA expression datasets of DR, DN, and DFU revealed total 681 upregulated genes [specific (AB).u], 884 downregulated genes [specific (AB).d], 11 upregulated miRNA, and 30 downregulated miRNA specific for DR (Figures 2, 3). Table 2 enlists all miRNA specific for DR. Further, among 681 up-regulated genes, 271 genes were also found to be hypo-methylated, and 78 were the targets of down-regulated miRNA, and among 884 down-regulated genes 84 genes were also hyper-methylated and 8 were the targets of up-regulated miRNA (Figure 4 and Supplementary Table 1).

**TABLE 2:** Differentially expressed DR specific miRNA.

Up-regulated miRNA	hsa-mir-320a, ssc-mir-24-2*, hsa-mir-320d-2, hsa-mir-455, hsa-mir-320d-1, tni-mir-23a-2*, tni-mir-23a-1*, ssc-mir-24-1*, hsa-let-7b, lca-mir-23a*, tni-mir-23a-3*
Down-regulated miRNA	hsa-mir-16-2, hsa-mir-486, hsa-mir-20b, hsa-mir-15b, hsa-let-7i, hsa-mir-16-1, hsa-mir-30e, hsa-mir-502, hsa-mir-17, hsa-mir-20a, hsa-mir-532, hsa-mir-18a, hsa-mir-222, hsa-mir-363, hsa-mir-194-2, hsa-mir-660, hsa-mir-194-1, hsa-mir-29a, hsa-let-7g, hsa-mir-130a, hsa-mir-27a, hsa-mir-192, hsa-mir-106b, hsa-mir-150, hsa-mir-106a, hsa-mir-25, hsa-mir-451, hsa-mir-15a, hsa-mir-30d, hsa-mir-126

*^∗^microRNA from species other than human. Human homologous of these miRNA were considered to find their targets.*

#### Potential DR Candidate Gene

A total of 40 potential DR candidate genes (genes showing altered expression as well as methylation pattern and were also the target of altered miRNAs) were identified. All of them were upregulated, hypomethylated, and targets of downregulated miRNA (Figure 4 and Supplementary Table 1).

### Genes Involved in Early Stage of DR

**FIGURE 5:**
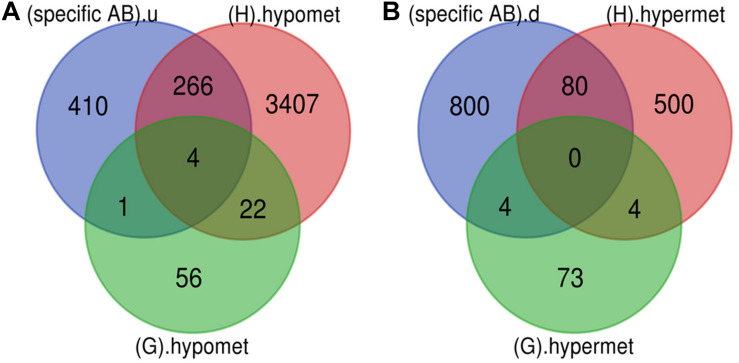
Early genes. **(A)** Up-regulated and hypomethylated genes: total 83 genes were hypomethylated during NPDR while 3699 during PDR out of which 271 genes were also up-regulated in DR cases. 5 up-regulated and hypomethylated genes were present in NPDR stage of DR. **(B)** Down-regulated and hypermethylated genes: total 81 genes were hypermethylated during NPDR while 584 during PDR out of which 84 genes were also downregulated in DR cases. 4 down-regulated and hypermethylated genes were present in NPDR stage of DR (specific AB).u: (up-regulated genes specific for DR); (G).hypomet: (hypomethylated genes in NPDR); (H).hypomet: (hypomethylated genes in PDR); (specific AB).d: (down-regulated genes specific for DR); (G).hypermet: (hypermethylated genes in NPDR); (H).hypermet: (hypermethylated genes in PDR).

Various pathological changes in retina of diabetic individual start even before the appearance of DR associated symptoms (Vujosevic et al., 2019). Thus DR remains asymptomatic during its initial stages, and by the time symptoms appear, the individual already suffers with some vision loss. The available treatment can preserve the remaining vision but can’t compensate for the already lost vision (Ellis et al., 2013). Further, metabolic memory phenomenon is believed to occur due to various epigenetic modifications occurring during the early stage of the disease (Intine and Sarras, 2012; Maghbooli et al., 2015). Hence, determining genes that play a critical role during early stage of the disease could help in preventing the disease progression during early stage of DR and could also help in finding a way to abolish metabolic memory phenomenon. We found a total of 9 genes showing differential expression and methylation pattern in the early stage of DR, i.e., NPDR out of which 5 (*NR1H4*, *ROCK2*, *HTATIP2*, *UHRF1*, and *NTM*) were upregulated-hypomethylated and 4 (*MAPT*, *FAM69C*, *FHOD3*, and *IGSF21*) were downregulated-hypermethylated genes (Figure 5, Table 3, and Supplementary Table 1). The identified early genes were found to be involved in one or more crucial events associated with DR progression like angiogenesis, oxidative stress, inflammation, etc. Further, some of the early genes like *ROCK2* (Koch et al., 2014; Lu et al., 2020), *UHRF1* (Ramesh et al., 2016), and *MAPT* (C. C. Zhang et al., 2016) are shown to participate in neurodegeneration, which is one of the earliest events in DR development. Also, in the present study, one of the identified early genes, i.e., *NR1H4*, was also found to be the target of one of the downregulated miRNAs (has-mir-192) identified in plasma sample of NPDR cases.

**TABLE 3:** List of probable DR associated genes.

Categories		Genes
Hub genes		*FN1* (Fibronectin 1), *IL6* (Interleukin 6), *COL1A2* (Collagen Type I Alpha 2 Chain), *COL4A1* (Collagen Type IV Alpha 1 Chain), *COL4A2* (Collagen Type IV Alpha 2 Chain), *SPARC* (Secreted Protein Acidic And Cysteine Rich), *MMP9* (Matrix Metallopeptidase 9)
Early genes	Upregulated and hypomethylated	*NR1H4* (Nuclear Receptor Subfamily 1 Group H Member 4), *ROCK2* (Rho Associated Coiled-Coil Containing Protein Kinase 2), *HTATIP2* (HIV-1 Tat Interactive Protein 2), *UHRF1* (Ubiquitin Like With PHD And Ring Finger Domains 1), *NTM* (Neurotrimin)
	Downregulated and hypermethylated	*MAPT* (Microtubule Associated Protein Tau), *FAM69C* (Family With Sequence Similarity 69 Member C), *FHOD3* (Formin Homology 2 Domain Containing 3), *IGSF21* (Immunoglobin Superfamily Member 21)

*Hub genes: potential candidate genes of DR which possess large number of interaction. Early genes: genes showing altered expression and methylation pattern during an early stage of DR.*

### PPI Network Construction, Hub Genes, and Module Identification

**FIGURE 6:**
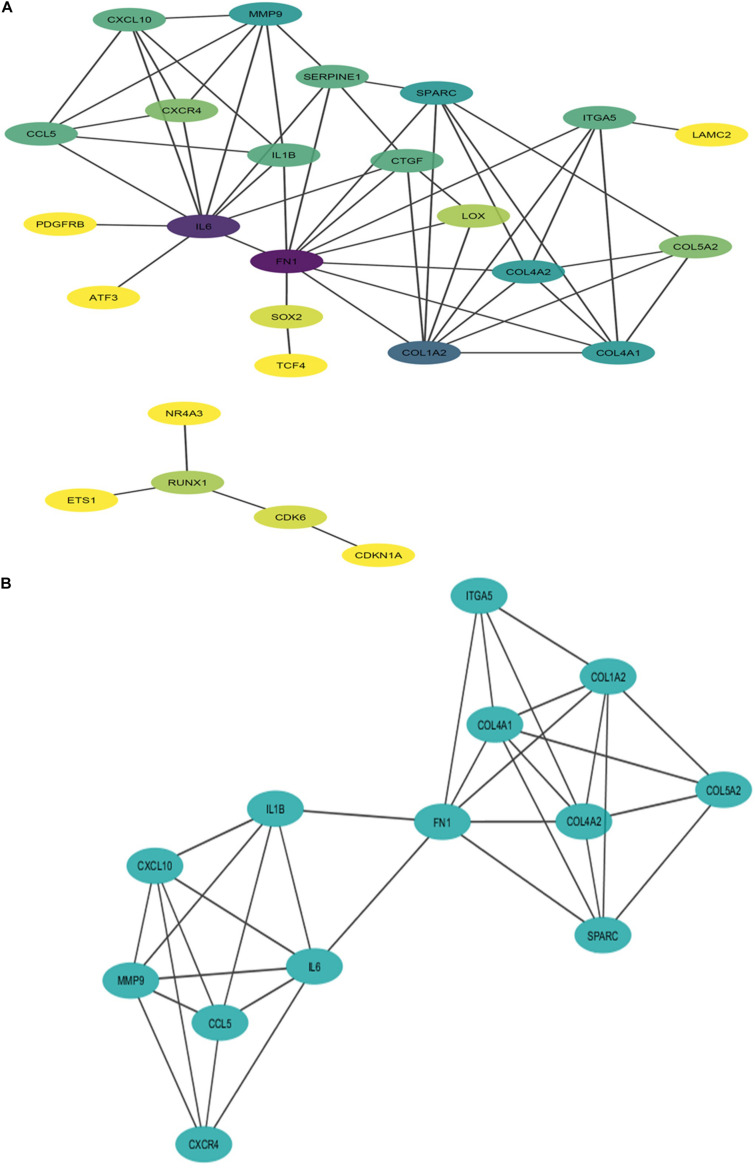
Interaction network and module of potential DR candidate genes. **(A)** Protein-protein interaction (PPI) network of potential DR candidate genes: the intensity of node color denotes the degree of interactions it has with other nodes (dark purple color denotes the highest number of interactions followed by blue, light blue, green, light green, etc., and yellow denotes the lowest number of interaction). **(B)** Module (MCODE score 5.67 and number of nodes 13) obtained from PPI network of potential DR candidate genes.

The PPI network of potential candidate genes showed a total of 26 interacting nodes with minimum interaction score of 0.7 (high confidence) (Figure 6A). MCODE detected 1 module having MCODE score 5.67 and number of node 13 (Figure 6B) while CytoHubba revealed 7 hub genes (*FN1*, *IL-6*, *COL1A2*, *COL4A1*, *COL4A2*, *SPARC*, and *MMP9*) (Table 3) with number of interactions >5 in the PPI network. DR specific miRNAs associated with hub genes are listed in Table 4. Most of the identified hub genes belong to the collagen group of extracellular matrix. Though evidence suggests involvement of extracellular matrix component in DR pathogenesis, not much study has been completed on collagen in association with DR. However, genes like *COL4A1* (Alavi et al., 2016), *COL4A2* (Alavi et al., 2016), and *FN1* (Moradipoor et al., 2016) are found to have association with the DR pathogenesis. Studies have also found *SPARC* (Fu et al., 2019), *IL-6* (Rojas et al., 2011), and *MMP9* (Kowluru et al., 2012) playing roles in DR development by affecting one or more factors responsible for DR like angiogenesis, inflammation, etc.

**TABLE 4:** Hub genes and the associated altered miRNA.

Hub genes*	miRNA*
*FN1*	hsa-let-7g
*IL6*	hsa-mir-451, has-mir-106a
*COL1A2*	hsa-let-7g, has-mir-25, has-mir-29a
*COL4A1*	hsa-mir-29a
*COL4A2*	hsa-mir-29a
*SPARC*	hsa-mir-29a
*MMP9*	hsa-mir-451, has-mir-15b

*^∗^All hub genes were up-regulated and their associated miRNAs were down-regulated.*

### Gene Ontology and KEGG Pathway Analysis

The gene ontology and KEGG pathway analysis revealed many biological processes, molecular functions, and pathways linked with potential candidate genes, hub genes, genes present in module, and some of the early genes of DR that can play an essential role in the pathogenesis of DR by regulating each other, enhancing pathological activities, and forming other cross communications.

The enrichment analysis revealed that the potential candidate genes, hub genes, and genes present in modules were enriched in molecular functions like binding to protein, organic cyclic compound, ions and extracellular matrix; hydrolase, oxidoreductase, transferase, and catalytic activity; transcription regulation; signaling receptor; enzyme and receptor regulation, etc. Further, the enriched biological processes were various cellular processes like signal transduction, movement of cells, cellular metabolic processes, cellular response to stimulus, regulation of various biological processes and molecular functions, immune response, leukocyte migration, oxidation-reduction process, metabolic processes, cell adhesions, etc. The enriched KEGG pathways were PI3K-Akt signaling pathway, ECM-receptor interaction, Focal adhesion, TNF signaling pathway, Toll-like receptor signaling pathway, Protein digestion and absorption, NOD-like receptor signaling pathway, Chemokine signaling pathway, etc. Some of the enriched GO and KEGG pathways of hub genes are shown in Table 5 while the list of probable DR associated enriched GO and KEGG pathways of potential candidate genes, hub genes, and genes present in module are provided in Supplementary Table 2.

**TABLE 5:** List of some of the enriched GO and KEGG pathways of hub genes.

Terms	GO/KEGG pathways	FDR value
Biological Processes (BP)	Extracellular structure organization (GO:0043062)	1.59E-06
	Endodermal cell differentiation (GO:0035987)	1.21E-03
	Cellular response to organic substance (GO:0071310)	1.27E-03
	Cellular response to chemical stimulus (GO:0070887)	2.88E-03
	Response to organic substance (GO:0010033)	3.11E-03
	Circulatory system development (GO:0072359)	4.70E-03
	Platelet activation (GO:0030168)	9.50E-03
	Collagen-activated tyrosine kinase receptor signaling Pathway (GO:0038063)	9.96E-03
	Blood vessel development (GO:0001568)	1.02E-02
	Positive regulation of cell migration (GO:0030335)	1.08E-02
Molecular Functions (MF)	Platelet-derived growth factor binding (GO:0048407)	7.15E-03
	Extracellular matrix structural constituent conferring tensile strength (GO:0030020)	7.24E-04
	Collagen binding (GO:0005518)	1.69E-03
	Extracellular matrix structural constituent (GO:0005201)	3.76E-06
	Structural molecule activity (GO:0005198)	1.48E-03
KEGG pathways	hsa04512:ECM-receptor interaction	0.018044733
	hsa04151:PI3K-Akt signaling pathway	0.028095395
	hsa05200:Pathways in cancer	0.047128876

The gene ontology and pathway analysis of individual hub genes and early genes showed that many of those genes were involved in biological processes, molecular functions, and pathways that are or can be associated with DR pathogenesis. For example, biological processes like angiogenesis, inflammatory response, neurogenesis, blood vessel development, extracellular matrix organization, etc.; molecular functions like protein binding, receptor binding, collagen binding, growth factor activity, extracellular matrix structural constituent, etc.; KEGG pathways like ECM receptor interaction, AGE-RAGE signaling pathways, PI3K-Akt signaling pathway, focal adhesion, etc. were associated with one or more genes. Supplementary Table 3 enlists the gene ontologies and KEGG pathways of individual hub genes and early genes based on their probable association with DR pathogenesis.

## Discussion

The complications associated with human health results from alterations in gene expression pattern, either by genetic, epigenetic modifications or other mechanisms. Today, the increased rate of diabetic incidences has also increased the rate of its associated complications, and diabetic retinopathy that affects approximately 34.6% of diabetic individuals (Yau et al., 2012) accounts for about 4.8% cases of blindness worldwide (Drake, 2007). Also available DR treatments suffer from one or more limitations such as economic burden, variability in drug response among patients, accessibility of the healthcare in rural areas, etc. Further, metabolic memory phenomena associated with diabetes has increased a great concern for early diagnosis and treatment strategies. Therefore, determining DR specific potential genes, genes altered during early stage of DR, their functions, molecular pathways, and interacting partners may lead to the finding of early diagnostic and better treatment methods. DNA methylation and miRNAs are among the various epigenetic modifications that are responsible for alterations in various genes expression during DR pathogenesis (Maghbooli et al., 2015; X. Zhang et al., 2017). Thus, they may play a crucial role in regulation of various biological processes, functions, and pathways associated with DR. Hence integration of gene expression profiling, gene methylation profiling, and miRNA expression profiling data could help in identification of more accurate and specific genes that may play an indispensable role in DR progression and pathogenesis.

Abnormal inflammation, oxidative stress, and neovascularization are the prime events responsible for vision loss in DR. The inflammatory responses like adhesion of leukocytes with endothelial cells and their migration toward the inflamed area aggravates the pathogenesis. Further neurodegeneration is another event observed during early stages of DR. Hence, the product of any genes whose pathways, functions, or processes affect these events either directly or indirectly can be involved in the disease progression.

Regarding the individual genes, we find that most of the hub genes belong to the collagen group of extracellular matrix. Studies have shown various extracellular matrix components to be involved in the development of DR, but only a few studies have been done on collagen in context to DR. There is not much study done on *COL1A2* (Collagen Type I Alpha 2 Chain) about DR. However, Type IV collagen, the major protein of basement membrane matrix, shows increase in its expression level in vitreous and probably also in serum with the duration of diabetes and is exalted in DR condition (Kotajima et al., 2001). Mutation in *COL4A1* (Collagen Type IV Alpha 1 Chain) and *COL4A2* (Collagen Type IV Alpha 2 Chain) has been found to elevate the risk of DR development by causing various abnormalities like vascular lesions, raising the expression of *Vegfa, Pdgfb*, and *Pgf* leading to neovascularization (Alavi et al., 2016). *COL4A1* is also associated with obesity, one of the risk factors of diabetes. Moreover, *FN1* (Fibronectin 1), a gene encoding fibronectin, is found to be up-regulated in T2DM and might be involved in angiogenesis, inflammatory response, and cell adhesion. Its level is increased in various tissues including retina, thus changing extracellular matrix (ECM) in endothelium and promoting damage to vessels wall. Also, endothelin- (ET-) dependent pathway is involved in the up-regulation of FN-1 during diabetes that involves activation of NF-kβ and AP1 transcription factors (Moradipoor et al., 2016). Further, IL-6 (Interleukin-6), which is a potent proinflammatory cytokine, plays an essential role in DR pathogenesis. Knockout of IL-6 resulted in reduced leukocytes adhesion in retinal blood vessels and TNF-alpha level in microglial cells of retina (Rojas et al., 2011). However, one study showed that IL-6 protects muller cells from glucose toxicity, thus playing a protective role in DR (Coughlin et al., 2019). *MMP9* (Matrix Metallopeptidase 9) encodes protein that is involved in the breakdown of extracellular matrix. It is involved in DR development and progression by accelerating apoptosis of retinal capillary cells in the early phase of DR and angiogenesis in the later phase (Kowluru et al., 2012). The level of MMP-9 differs with the stages of DR and was found to contribute more than MMP-1 in DR pathogenesis (Kwon et al., 2016). Various histone modifications, DNA methylations, and their role in metabolic memory formation (Mishra and Kowluru, 2016; Kumari et al., 2020) are reported for *MMP9* during hyperglycemic conditions. *SPARC* (Secreted Protein Acidic And Cysteine Rich), a gene that encodes cysteine-rich acidic matrix-associated protein, is also involved in the development of DR. Retinal basement membrane of Type2DM patients showing thickening and permeability changes is found to secrete the protein encoded by *SPARC* (Watanabe et al., 2009). Further, *SPARC* was also found to mediate cellular adhesion, cell migration, and angiogenesis.

Moving to genes altered in early stage of DR, except for one study on *IGSF21* (Immunoglobin Superfamily Member 21) (Lin et al., 2016), none of the genes have been studied in context to DR. However, *ROCK2* (Rho Associated Coiled-Coil Containing Protein Kinase 2) (Koch et al., 2014; Lu et al., 2020), *UHRF1* (Ubiquitin Like With PHD And Ring Finger Domains 1) (Ramesh et al., 2016), and *MAPT* (Microtubule Associated Protein Tau) (C. C. Zhang et al., 2016) are shown to be associated with neurodegeneration. As neurodegeneration has been observed as one of the earliest events in the onset of DR, these genes might be responsible for retinal pathological changes in early DR and might also play a role in metabolic memory formation. Additionally, there are so many studies done on *ROCK1* but not on *ROCK2*. However, ROCK has an essential role in the pathogenesis of DR. It affects the expression and function of adhesion molecules and its inhibitor significantly reduced this adhesion process by reducing the activation of ROCK. ROCK pathway also plays a critical role in angiogenesis (Arita et al., 2010). Moreover, abnormal ROCK pathways are responsible for various neurological disorders. In one study ROCK inhibitor was shown to increase the regeneration of retinal ganglion cell (Lingor et al., 2007). Another study showed increase in ROCKII protein level in NMDA-induced retinal neurotoxicity, and its inhibitor acted as neuroprotective agent by abolishing the increase in ROCKII level (Kitaoka et al., 2004). Apart from this, *UHRF1*, which encode a protein that regulates DNA and histone methylation, and *NR1H4* (Nuclear Receptor Subfamily 1 Group H Member 4), which encode ligand-activated transcription factor, are also found to be linked with inflammation (Fiorucci et al., 2010; Wang et al., 2018), oxidative stress (Gai et al., 2017; J. K. Kim J. K. et al., 2020), and angiogenesis (Guo and Mo, 2020). Interestingly, in the present study, *NR1H4* was also found to be one of the targets of down-regulated miRNA identified in a plasma sample of NPDR cases and thus can act as a preferred candidate in studies concerned with identification of circulatory prognostic biomarker for DR.

This study revealed many hub genes and few early genes that have the potential to act as a target in future DR research, but this study suffers from its own limitations. During the selection of probable candidate genes in the early stage of DR (early genes), only gene methylation dataset was taken into consideration due to unavailability of NPDR samples in the gene expression (mRNA expression) dataset, and this is one of the major limitations of this study. The miRNA data was also not considered here because targets of miRNAs are diverse, which may include many unrelated genes and therefore may decrease the specificity of the identified genes to the particular context.

Further, as different datasets differ in the source of population for sample collection, and also in some cases single dataset contains sample from different parts of body, integration of these may lead to heterogeneity and affect the results. However, by maintaining the statistical significance of data, the integration of multiple datasets can be beneficial in capturing multiple molecular alterations, thus improving the prediction accuracy while the presence of diverse source of population and different regions of body can also lead to identification of potential global candidate genes for the disease.

Also, not sufficient studies are available for the identified genes with respect to DR. Furthermore, validation of the obtained results is necessary for the genes to be considered as a representative gene for DR.

## Conclusion

Diabetic retinopathy is a consequence of multiple altered metabolic processes, biological functions, and pathways that are linked among themselves in one or the other ways, and these alterations are in turn associated with one or more altered expression of genes.

The study identified 7 hub genes (*FN1*, *IL-6*, *COL1A2*, *COL4A1*, *COL4A2*, *SPARC*, and *MMP9*) that could play a potential role in the aggravation of DR pathogenesis. Further, some of the early genes like *NR1H4* and those participating in neurodegeneration (*ROCK2*, *UHRF1*, and *MAPT*) could be responsible for early pathological changes in DR and formation of metabolic memory and can be used as a potential prognostic biomarker and early therapeutic targets for DR.

## Data Availability Statement

The data of gene expression profiling, gene methylation profiling, and miRNA expression profiling were obtained from Gene Expression Omnibus (GEO) datasets available at the National Center for Biotechnology Information (NCBI) (https://www.ncbi.nlm.nih.gov/gds): GSE60436, GSE57362, GSE140959, GSE1009, GSE51674, GSE80178, and GSE84971.

## Author Contributions

NK, SC, and SG gave equal contribution in framing research topics, collecting study materials, performing the entire research work, analyzing the data, preparing the manuscript, proofreading, and so forth. AK wrote the introduction section of the manuscript and was involved in the data analysis. All authors read and approved the final manuscript.

## Conflict of Interest

The authors declare that the research was conducted in the absence of any commercial or financial relationships that could be construed as a potential conflict of interest.

## Footnotes

1 https://www.ncbi.nlm.nih.gov/gds

2 https://www.ncbi.nlm.nih.gov/geo/geo2r/

3 https://string-db.org/

4 https://david.ncifcrf.gov/

5 https://www.ebi.ac.uk/QuickGO/

6 https://www.genome.jp/kegg/pathway.html

## Abbreviations

DEGs: differentially expressed genes
DFU: diabetic foot ulcer
DMGs: differentially methylated genes
DN: diabetic nephropathy
DR: diabetic retinopathy
FVM: fibro-vascular membrane
NPDR: non-proliferative diabetic retinopathy
NV: neovascularization
PDR: proliferative diabetic retinopathy.
