# Supplementary material for: Integrative Computational Approach Revealed Crucial Genes Associated With Different Stages of Diabetic Retinopathy
Source: Front Genet. 2020 Nov 12;11:576442. doi: 10.3389/fgene.2020.576442 (PMC7693709; doi:10.3389/fgene.2020.576442)
Supplement: Supplementary file 1 [file Table_1.DOCX]

**Table S1: Summarization of total number of genes identified in various sets of study**

| **Sets of study** | | **Number of genes** | |
| --- | --- | --- | --- |
|  |  | **Up-regulated /**  **Hyper-methylated** | **Down-regulated /**  **Hypo-methylated** |
| **Diabetic retinopathy (DR)** | Gene expression profiling | 743 (Total)  681 (DR specific) | 971 (Total)  884 (DR specific) |
|  | Gene methylation profiling | 81 (NPDR)  584 (NPDR) | 83 (NPDR)  3699 (PDR) |
|  | miRNA expression profiling | 11 (Total)  11 (DR specific) | 30 (Total)  30 (DR specific) |
| **Diabetic nephropathy (DN)** | Gene expression profiling | 855 | 408 |
|  | miRNA expression profiling | 126 | 35 |
| **Diabetic foot ulcer (DFU)** | Gene expression profiling | 353 | 864 |
|  | miRNA expression profiling | 27 | 1 |
| **Differentially expressed DR specific genes which were also differentially methylated** | | 271 (up-regulated & hypo-methylated)  84 (down-regulated & hyper-methylated) | |
| **Differentially expressed DR specific genes which were also the targets of differentially expressed DR specific miRNAs** | | 78 (up-regulated & targets of down-regulated miRNAs)  8 (down-regulated & targets of up-regulated miRNAs) | |
| **Potential DR candidate genes** | | 40 (up-regulated, hypo-methylated, targets of down-regulated miRNAs) | |
| **Early genes of DR** | | 5 (up-regulated, hypo-methylated)  4 (down-regulated, hyper-methylated) | |
| **Hub genes for DR** | | 7 (up-regulated, hypo-methylated, targets of down-regulated miRNAs) | |
| **Module** | | 13 (up-regulated, hypo-methylated, targets of down-regulated miRNAs) | |

**Potential DR candidate genes:** genes showing altered expression as well as methylation pattern and are also the target of altered miRNAs

**Hub genes:** potential candidate genes of DR which possess large number of interaction

**Early genes:** genes showing altered expression and methylation pattern during an early stage of DR
